# Lentinan-functionalized Selenium Nanoparticles target Tumor Cell Mitochondria via TLR4/TRAF3/MFN1 pathway

**DOI:** 10.7150/thno.46467

**Published:** 2020-07-11

**Authors:** Hui-Juan Liu, Yuan Qin, Zi-Han Zhao, Yang Zhang, Jia-Huan Yang, Deng-Hui Zhai, Fang Cui, Ce Luo, Man-Xi Lu, Piao-Piao Liu, Heng-Wei Xu, Kun Li, Bo Sun, Shuang Chen, Hong-Gang Zhou, Cheng Yang, Tao Sun

**Affiliations:** 1 State Key Laboratory of Medicinal Chemical Biology and College of Pharmacy, Nankai University, Tianjin, China.; 2 Tianjin Key Laboratory of Early Druggability Evaluation of Innovative Drugs and Tianjin Key Laboratory of Molecular Drug Research, Tianjin International Joint Academy of Biomedicine, Tianjin, China.; 3 Department of Gastroenterology and Hepatology, General Hospital, Tianjin Medical University, Tianjin Institute of Digestive Disease, Tianjin, China.; 4 Department of Anesthesiology, Tianjin Fourth Central Hospital, Tianjin, China.

**Keywords:** malignant ascites, ovarian cancer, lentinan, selenium nanoparticles, mitochondria targeting pathway

## Abstract

**Rationale:** Malignant ascites caused by cancer cells results in poor prognosis and short average survival time. No effective treatment is currently available for malignant ascites. In this study, the effects of lentinan (LNT)-functionalized selenium nanoparticles (Selene) on malignant ascites were evaluated. Furthermore, the mechanism of Selene targeting mitochondria of tumor cells were also investigated.

**Methods:** Selene were synthesized and characterized by TEM, AFM and particle size analysis. The OVCAR-3 and EAC cells induced ascites models were used to evaluate the effects of Selene on malignant ascites. Proteomic analysis, immunofluorescence, TEM and ICP-MS were used to determine the location of Selene in tumor cells. Mitochondrial membrane potential, ROS, ATP content, and caspase-1/3 activity were detected to evaluate the effect of Selene on mitochondrial function and cell apoptosis. Immunofluorescence, Co-IP, pull-down, duolink, Western blot, and FPLC were used to investigate the pathway of Selene targeting mitochondria.

**Results:** Selene could effectively inhibit ascites induced by OVCAR-3 and EAC cells. Selene was mainly located in the mitochondria of tumor cells and induced apoptosis of tumor cells. The LNT in Selene was involved in caveolae-mediated endocytosis through the interaction between toll-like receptor-4 (TLR4) and caveolin 1 (CAV1). Furthermore, the Selene in the endocytic vesicles could enter the mitochondria via the mitochondrial membrane fusion pathway, which was mediated by TLR4/TNF receptor associated factor 3 (TRAF3)/mitofusin-1 (MFN1) protein complex.

**Conclusion:** Selene is a candidate anticancer drug for the treatment of malignant ascites. And TLR4/TRAF3/MFN1 may be a specific nano-drug delivery pathway that could target the mitochondria.

## Introduction

Ascites caused by malignant tumor is called malignant ascites. The invasion of tumor tissue into peritoneum or abdominal cavity can lead to the damage of vascular endothelial cells and the increase of vascular permeability 1. The main causes of ascites are the alteration of the microvascular networks' permeability and lymphatic obstruction 2, 3. Malignant tumors, such as ovarian and colon cancers, could secrete vasoactive factors that alter the microvascular network permeability and lead to transudative and exudative ascites 4, 5. Ovarian malignancy is the most common cause (37%) of malignant ascites, which leads to poor prognosis and short average survival time 6. Several methods, such as intraperitoneal (i.p.) hyperthermic perfusion chemotherapies, diuresis and abdominal puncture drainage, are commonly used for the clinical treatment of malignant ascites. However, chemotherapy exhibits severe side effects and can't effectively inhibit inflammatory cytokines; it may even promote recurrence and inflammatory cytokine storms.

Pyroptosis is a kind of necrotic and inflammatory-programmed cell death induced by inflammatory caspases 7. Pyroptosis is accompanied by a large number of inflammatory cytokines that may stimulate ascites production 8. Chemotherapeutic drugs could induce pyroptosis of tumor cells 9-11. This phenomenon may explain why ascites rebound after chemotherapy. By contrast, mitochondrion-mediated apoptosis is an immunologically silent process that may be helpful for clearing tumor cells and ascites 12. Thus, inducing apoptosis rather than pyroptosis of the cancer cells is an important approach to inhibit malignant ascites.

Lentinan (LNT) is a special type of β-glucan obtained from *Lentinus edode* and has a wide variety of biological and physiological activities 13. LNT is used in clinics for the treatment of malignant ascites in China, which has high security but low efficiency 14, 15. Selenium (Se) has a potential application in cancer therapy due to its excellent biological activity and low toxicity 16, 17. Moreover, the biocompatibility and efficacy of Se nanoparticles (SeNPs) are better than those of inorganic and organic Se compounds 18-21. However, the effect of SeNPs in malignant ascites has not been reported. LNT could inhibit inflammatory cell infiltration and the transformation of inflammation and cancer by targeting toll-like receptor-4 (TLR4), which is highly expressed in tumor cells and tumor-infiltrating immune cells 14, 22. Previous studies have also shown that LNT could contribute to the stable dispersion of SeNPs in water 23.

On the basis of the above research, LNT-functionalized SeNPs (hereinafter referred to as Selene) may exhibit anti-inflammatory and apoptosis-inducting effects, which could contribute to the treatment of malignant ascites. Thus, in this work, the effect of Selene on malignant ascites was assessed. The results showed that Selene could efficiently inhibit the ascites in the Ehrlich ascites cancer (EAC) and OVCAR-3 malignant ascites models. Although SeNPs could affect the mitochondrial function, nanoparticles are mainly degraded in the lysosome, which may induce cell pyroptosis and necroptosis 24, 25. Inflammatory cytokines caused by pyroptosis and necroptosis may reduce the efficacy of ascites treatment. Therefore, developing a new kind of functionalized SeNPs for malignant ascites treatment that could selectively enter the mitochondria and escape from lysosomes is an important strategy. This study demonstrated that Selene could efficiently target the mitochondria via the TLR4/TNF receptor-associated factor 3 (TRAF3)/mitofusin (MFN1) pathway, indicating that TLR4/TRAF3/MFN1 may be a specific nanodrug delivery pathway targeting the mitochondria.

## Methods

### Materials

Mitochondrial membrane potential assay kit (C3601), Fast silver staining kit (P0017S), and cell mitochondria isolation kit (C2006) were purchased from Beyotime (Shanghai, China). Reactive oxygen species (ROS) assay kit (KGT010-1) and 3-(4,5-Dimethylthiazol-2-yl)-2,5-diphenyl tetrazolium bromide (KGA312) were purchased from KeyGen Biotech (Nanjing, China). Duolink In Situ Probemaker (DUO92010/DUO92009) and Duolink flowPLA detection kit-Red (DUO94001) were purchased from Sigma-Aldrich (Saint Louis, USA). All siRNA and shRNA were purchased from Origene (Beijing, China). Anti-OPA1 [DF8587, 1:500 for Western blot (WB), 1/100 for immunofluorescence (IF), and 1:200 for immunoprecipitation (IP)], anti-TRAF3 (DF7181, 1:1000 for WB, 1:100 for IF, and 1:200 for IP), anti-MFN1 (DF7543, 1:1000 for WB, 1:100 for IF, and 1:200 for IP), anti-TLR4 (AF7017, 1:1000 for WB, 1:100 for IF, and 1:200 for IP) monoclonal antibodies and goat anti-rabbit/mouse-horseradish peroxidase-conjugated secondary antibody (S0001/S0002, 1:5000 dilution) were purchased from Affinity (Cincinnati, USA).

### Preparation of Selene

Selene was mainly prepared following the procedure described by Jia *et al.* 23 with some modification. In brief, freshly prepared LNTs (2 mg/mL) in aqueous solution (20 mL) were denatured into single chains at 140 °C. After cooling to room temperature, the LNT solution was mixed with 500 μL of selenite sodium (0.1 M) and stirred for 5 min. Approximately 1 mL of ascorbic acid aqueous solution (0.2 M) and 20 μL of glutaraldehyde (50%) solution was added dropwise to the resulting mixture, which was then stirred for 24 h at room temperature. The reacted product was dialyzed using regenerated cellulose tubes (Mw cutoff of 8000) against ultrapure water for 2 days. Then, the resulting solution was freeze dried using a lyophilizer to obtain SeNPs/LNT composites (red powder, Selene). The product was mixed with 6-coumarin to obtain green fluorescent Selene.

### Characterization of Selene

The morphology of the diluted Selene solution was observed using transmission electron microscopy (TEM, FEI-Tecnai G2 Spirit TWIN). Samples for TEM observation were obtained by dropping the solutions onto C-coated Cu grids without any purification, nor inspissation. The elemental composition of Selene was detected using the spectrometric system of the TEM instrument. The size and 3D structure of the nanoparticles were confirmed using atomic force microscopy (AFM, Bruker, Dimension Icon). The zeta potential of the nanoparticles was measured using a Nano-ZS ZEN3600 (Malvern Instruments, UK) instrument at 25 °C. The Fourier transform infrared spectroscopy (FTIR) spectra of Selene and LNT were analyzed using a spectrophotometer (PerkinElmer, Spectrum65). The samples were blended with KBr powder, pressed into pellets, and scanned within the range of 4,000-400 cm^-1^.

### Cell lines and cell culture

OVCAR-3 and EAC cells were purchased from KeyGen Biotech (Nanjing, China). The cells were cultured in RPMI 1640 medium supplemented with 10% fetal calf serum and 1% penicillin/streptomycin at 37 °C and 5% CO_2_ in a humidified chamber.

### Reactive Oxygen Detection

OVCAR-3 cells were inoculated into six-well plates and cultured at 37 °C overnight. Then, they were treated with Selene or the solvent control for 4 h. The cell culture medium was removed, and an appropriate volume of diluted DCFH-DA was added in the cells. The volume of the DCFH-DA should fully cover the cells. Then, the cells were incubated at 37 °C in the incubator for 50 min. Subsequently, the cells were washed with serum-free cell culture medium three times to remove the residual DCFH-DA. Finally, the cells were collected and detected using flow cytometry (Guava EasyCyte, Millipore, USA).

### Mitochondria separation

The cells were collected and washed with pre-cooled PBS. A mitochondrion separation reagent (1.5 mL) with PMSF (1 mM) was added to 3 × 10^7^ cells and incubated for 10-15 min on ice. The cell suspension was then transferred to a glass homogenizer and homogenized for approximately 30 times. The cells were centrifuged at 600× g under 4 °C for 10 min. Then, the supernatant was carefully transferred to another tube and centrifuged at 11,000× g under 4 °C for 10 min, and the precipitate was isolated mitochondria.

### Immunofluorescence assay

OVCAR-3 cells cultured on climbing films in 24-well plate were treated with Selene and SeNPs. Then, the cells were fixed in 4% paraformaldehyde for 15 min at room temperature and incubated in 0.01% Triton X-100 solution for 15 min. Subsequently, the cells were blocked with 5% BSA for 1.5 h. The primary antibody diluted with 1% BSA (1:200) was added in the cells, which were then incubated at 4 °C overnight. After the cells were washed with PBS, they were incubated with fluorescently conjugated secondary antibodies (1:200, KeyGen Biotech) for 1 h. The samples were counterstained with mounting medium containing DAPI (Solarbio, China) for 2 min. Finally, the cells were observed under a confocal microscope (Nikon, Japan).

### Western-blot analysis

The total proteins isolated from OVCAR-3 cells or the proteins isolated using IP beads were separated via SDS-polyacrylamide gel electrophoresis (PAGE) and then transferred to a PVDF membrane (Millipore, USA). The membranes were incubated for 2 h in TBST buffer contain 5% skim milk. The membranes were then incubated with primary antibodies. After the primary antibody was washed out using TBST, the PVDF membrane was further incubated with horseradish peroxidase (HRP)-labeled secondary antibodies. Finally, the target proteins were visualized using enhanced chemiluminescence substrate reagents (Millipore, USA).

### Assessment of Selene internalization in OVCAR-3 cells

The cells were incubated with 0.1% (w/v) sodium azide (NaN_3_) and 50 mM 2-deoxyglucose (DOG) 26 for 1 h before the addition of Selene (10 μM) to detect whether the uptake of Selene into the OVCAR-3 cells was energy dependent. The effect of temperature on Selene uptake was examined after pre-incubation of cells for 1 h at 4 °C. Then, the cells were incubated with Selene (10 μM) for 1 h at 4 °C. OVCAR-3 cells were treated with clathrin-mediated endocytic inhibitors (0.45 M sucrose or 20 mM chlorpromazine hydrochloride), caveolin-mediated endocytic inhibitors (5 mM methyl-β-cyclodextrin or 54 µM nystatin) and pinocytic inhibitors (EIPA 10 µM or amiloride 2.5 mM) in normal culture medium for 1 h at 37 °C to further clarify the specific endocytic pathways of Selene 27. Selene was then added to the cells and incubated for 1 h. Subsequently, the cells were washed three times with PBS, and the Se content in cells were analyzed by ICP-MS.

### Proximity ligation assay (PLA)

Duolink PLA was conducted as described in the literature to determine the proteins interactions 28. In brief, the OVCAR-3 cells were cultured on climbing films in 24-well culture plate overnight. The cells were fixed in 4% paraformaldehyde for 10 min and then washed with PBS. The cells were blocked with 5% BSA for approximately 45 min. Primary antibodies (1:200) diluted in blocking solution were added to the cells, which were then incubated overnight in a humid chamber at 4 °C. The cells were washed with washing buffer A before the addition of heterologous secondary antibody with oligonucleotide PLA probe. Subsequently, the cells were incubated with heterologous secondary antibody for 1 h at 37 °C in a wet room, incubated with diluted ligase (1:40) for 40 min at 37 °C and then incubated with diluted amplification solution (1:80) for 90 min at 37 °C after washing with wash buffer A. Finally, the cells were mounted using Duolink in situ mounting medium with DAPI and observed under the confocal microscope. The PLA signals were recognized as red fluorescent spots.

### Co-IP assay

Protein A/G beads were incubated with the antibody of the target protein or IgG (negative control) for 6-8 h at 4 °C to form the immune complex. Afterward, 1 × 10^7^ OVCAR-3 cells were lysed using 1 mL of RIPA lysis buffer. Then, the protein A/G beads were incubated with cell lysate overnight at 4 °C and washed with PBS containing 0.1% Nonidet P-40 (NP-40). Finally, the immune complex was eluted using the elution buffer, and the elution sample was analyzed using WB.

### Pull-down assay

Fresh OVCAR-3 cells (1 × 10^8^) expressing Flag-TLR4 were lysed using lysis buffer (0.2 mM EDTA, 50 mM Tris-HCl, pH 7.4, 150 mM NaCl) containing 0.3% NP-40 and protease inhibitor cocktail. Anti-FLAG tag beads were incubated with the cell lysate overnight at 4 °C. The beads were washed with lysis buffer containing 0.1% NP-40. Approximately 1 mL of 20% Flag peptide was added to the beads and incubated overnight at 4 °C to elute the Flag protein complex. The beads were centrifuged for 5 min at 13,000 rpm, and the supernatant was added to the 10 kDa ultrafiltration concentration tube and concentrated to 60 µL. Subsequently, 5× loading buffer was added to the eluents and heated for 10 min at 99 °C. The eluents were visualized on 10% SDS-PAGE, followed by silver staining with Silver Stain Kit (Beyotime). The differential protein bands were analyzed using mass spectrometry.

### Proteomic analysis

The OVCAR-3 cells treated with Selene were harvested, lysed and isolated by the SDS-PAGE. The protein bands were then digested with trypsin for 20 h at 37 °C, evaporated, and resuspended in 0.1% formic acid for LC-MS/MS analysis. The protein expression profile was detected using mass spectrometry. The differentially expressed proteins, which were significantly regulated (|logFC| > 1.5) in the samples treated with Selene, were analyzed using the Metascape website (http://metascape.org/) for Gene Ontology (GO) and Kyoto Encyclopedia of Genes and Genome (KEGG) enrichment analysis. The PPI network was analyzed on the STRING website (www.string-db.org/) and Cytoscape software.

### Animal research

The mice were obtained from the Animal Center Academy of the Military Medical Science (Beijing, China). All animals were kept under specific pathogen-free conditions. All animal procedures were conducted in accordance with the guidelines of the Animal Ethics Committee of the Tianjin International Joint Academy of Biotechnology and Medicine. The EAC cells were suspended in normal saline (1 × 10^7^ cells/mL), and 100 μL of cells were inoculated to the Kunming mice via intraperitoneal (i.p.) injection to establish EAC xenografts. The mice were divided into three sets: 1) drugs were administered from the fourth day after the injection of cancer cells; 2) drugs were administered from the seventh day after injection of cancer cells, when the ascites grew; and 3) the ascites were completely removed after they grew on day 7, and then the drug was administered. Each set was divided into five groups (n = 5): the normal control group, the EAC xenograft model group, and the drug administrated groups (3 mg/kg/day of Selene, 10 mg/kg/day of LNT, and 3 mg/kg/day of SeNPs).

Ovarian ascites BALB/c nude xenografts were established via i.p. injection of 100 μL of OVCAR-3 cells (1 × 10^7^ cells/mL). The drug was administered from the fourth day after the modeling, and the animals were randomly divided into five groups (n = 5), which was similar with the EAC xenografts. The B-ultrasound scanner for animals was used to detect the volume of ascites, which was reflected by the echo dark area in the abdominal cavity. Twenty days after the modeling, Evans blue was injected into the tail vein of the mice to measure the permeability of their abdominal wall membrane. After mice were euthanized, the ascites was collected, and the cells in the ascites were harvested and counted. The proteomics of cells were then determined.

### Cytokine antibody chip assay

The cytokine in the ascites was detected using mouse inflammation array (Raybiotech, USA). The glass chip was balanced using the buffer at room temperature for 0.5 h and dried for 1 h. The cytokine standard was diluted using the sample diluent. The chip was blocked using 100 μL of the sample diluent at room temperature for 1 h. Afterward, the diluent buffer was removed, and 100 μL of the samples and standards were added to the chip and incubated overnight at 4 °C. After the chip was cleaned using the diluent buffer, 80 μL of biotin-labeled detection antibody was added into the chip and incubated for 2 h. Subsequently, the chip was washed and incubated with 80 μL of Cy3-labeled streptavidin in the dark for 1 h. Finally, a laser scanner was used to detect the corresponding signal of each cytokine, and QAM-INF-1 software was used to analyze the data.

### Statistical analysis

All data were expressed as mean ± standard deviation (SD). Comparisons between groups were performed using ANOVA, followed by the Bonferroni post hoc test on SPSS software package (version 19.0, SPSS Inc., Chicago, IL, USA) and GraphPad software (version 7, GraphPad Software, Inc., La Jolla, CA, USA). The level of significance was set at *P* < 0.05.

## Results

### Preparation and characterization of Selene

**Figure 1:**
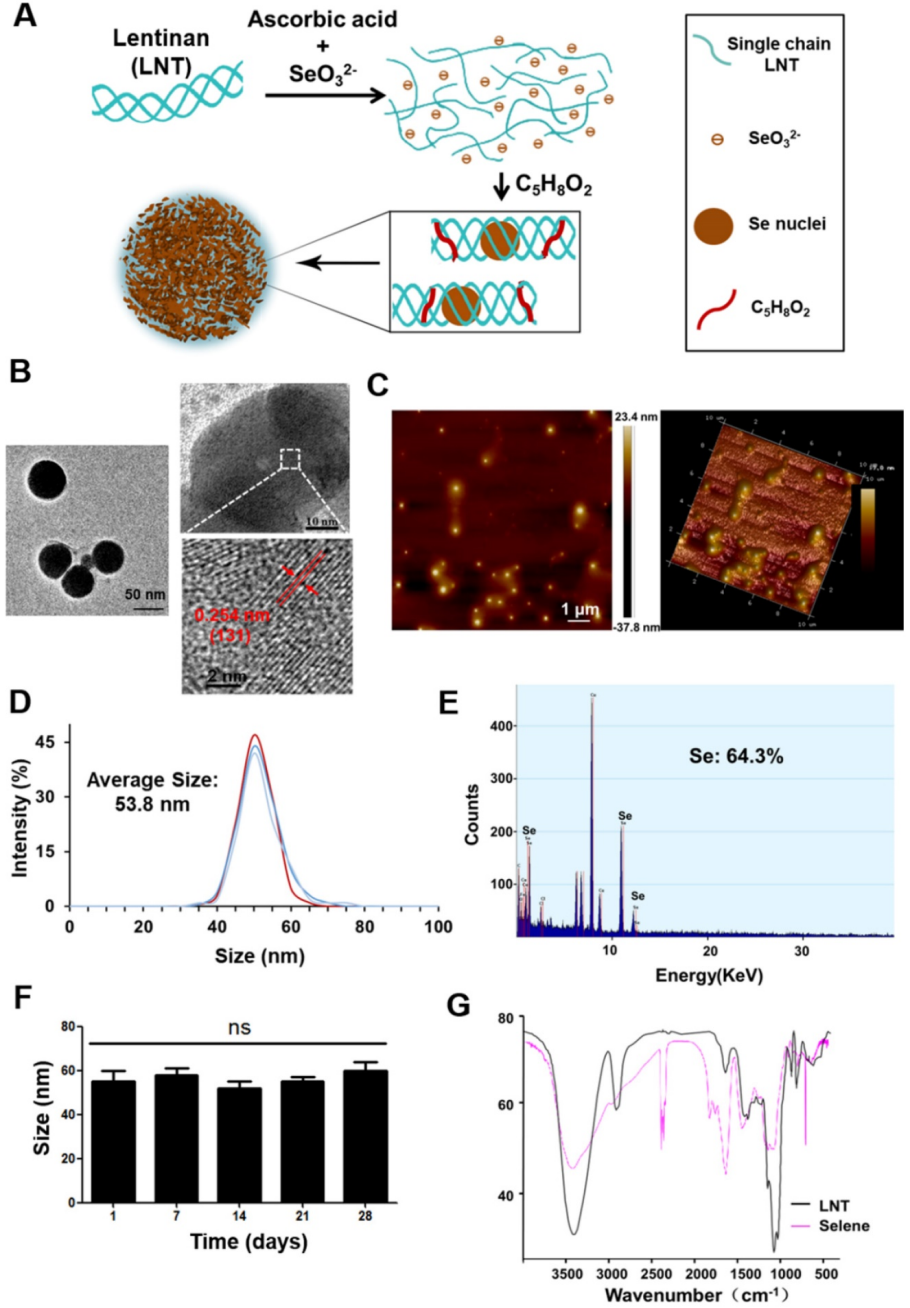
** Preparation and characterization of Selene. A.** Synthetic process of lentinan-functionalized Se nanoparticles (Selene). **B.** TEM images of Selene. **C.** AFM images of Selene. **D.** Size distribution of Selene. **E.** EDX detection results of Selene. **F.** Stability detection results of Selene in saline (0.9% NaCl) at room temperature. **G.** FTIR spectra of Selene. Results are shown as means ± SD (*P < 0.05, **P < 0.01).

In this study, Selene was synthesized under optimized conditions (Figure 1A) and characterized using TEM, AFM, energy dispersive X-ray (EDX), and FTIR. TEM and AFM results showed that the particle morphology of Selene presented monodisperse and homogeneous spherical structures with equable size (Figures 1B and 1C). The distance between the parallel lattice planes of Selene was 0.254 nm at room temperature. The average particle diameter of Selene was 53.8 nm (Figure 1D). EDX results showed that Se was the main constituent of Selene (Figure 1E). The Selene particles could remain stable for at least 28 days (Figure 1F) in physiological saline (0.9% NaCl). LNT and Selene showed different FTIR spectra (Figure 1G), and the characteristic absorption peak of the -OH groups in Selene was broadened, which indicates hydrogen bonding interactions between -OH groups on the LNT chain and Se. The characterization of SeNPs was shown in supplementary materials (Figure S1). Compared with Selene, SeNPs have a larger particle size and are easier to aggregate.

### Selene inhibited ascites induced by EAC* in vivo*

**Figure 2:**
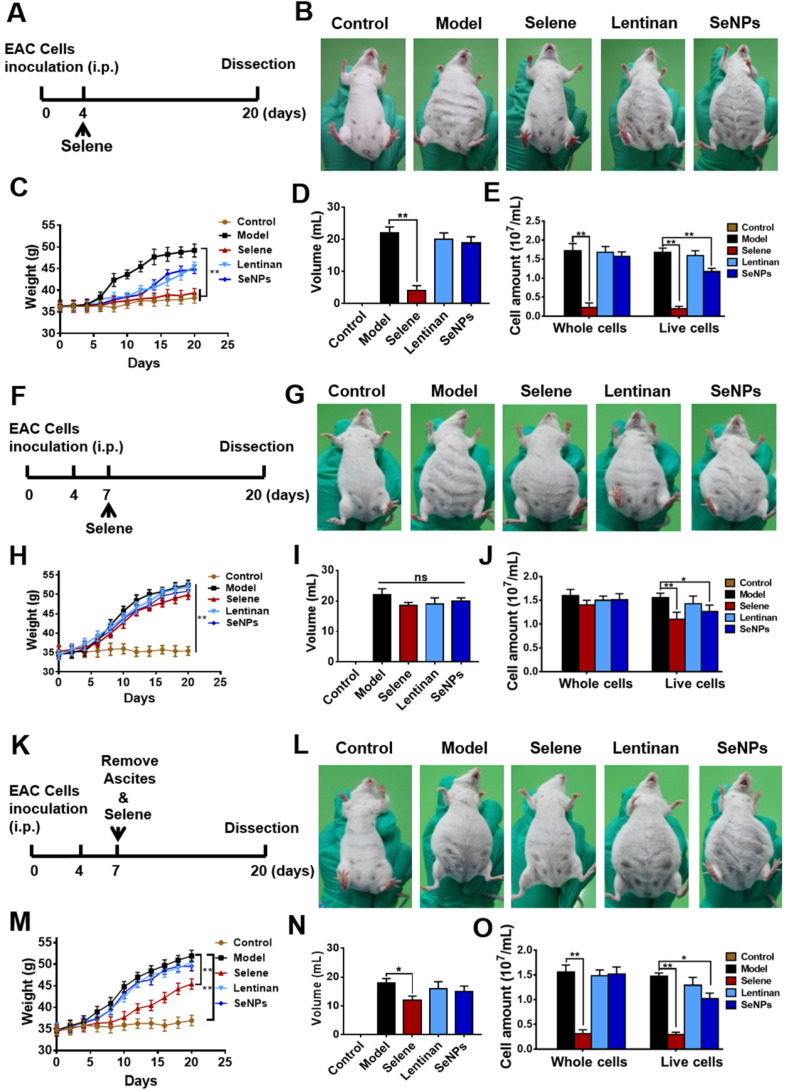
** Selene inhibited ascites induced by EAC* in vivo*. A.** Schematic diagram of model establishment and drug administration. The mice were treated with Selene, lentinan, or SeNPs from the fourth day after the modeling, when ascites began to appear. **B.** Representative images of mice in the different groups. **C-E.** Body weight, volume of ascites, and cancer cell numbers in ascites of each group. **F.** Schematic diagram of model establishment and drug administration. The mice were treated with Selene from the seventh day after the modeling, when a large amount of ascites appeared. **G.** Representative images of mice in different groups. **H-J.** Changes in body weight, volume of ascites, and cancer cell number in ascites of different groups. **K.** Schematic diagram of model establishment and drug administration. The mice were treated with Selene after the ascites were removed from the seventh day after the modeling. **L.** Representative images of mice in the different groups. **M-O.** Changes in body weight, volume of ascites, and cancer cell numbers in ascites of the different groups. Results are shown as means ± SD (*P < 0.05, **P < 0.01).

EAC malignant ascites model was established using BALB/c mice to explore whether Selene could suppress the ascites. The mice were administered with Selene from the fourth day after the EAC model was established (Figure 2A), when the ascites began to appear. Images of the mice in different groups showed that Selene could inhibit the ascites of the EAC model (Figure 2B). In addition, Selene could decrease the body weight (Figure 2C), the volume of ascites (Figure 2D), and EAC cell numbers (Figure 2E). The body weight and volume of ascites in the LNT- and SeNPs-treated groups were slightly affected. In another set of experiment, Selene was administrated via i.p. injection from the seventh day after the EAC model were established, when a large amount of ascites appeared (Figure 2F). The results showed that Selene could not suppress the ascites under this condition (Figures 2G-2J). Selene was also administered to mice via i.p. from the seventh day after removing the ascites (Figure 2K). The body weight, the volume of ascites, and EAC cell numbers were inhibited in Selene treated groups (Figures 2L-O). These results indicated that the best time to administer Selene was when the ascites first appeared. When a large amount of ascites appeared, the ascites should be removed before Selene administration.

### Selene inhibited ascites induced by ovarian cancer *in vivo*

**Figure 3:**
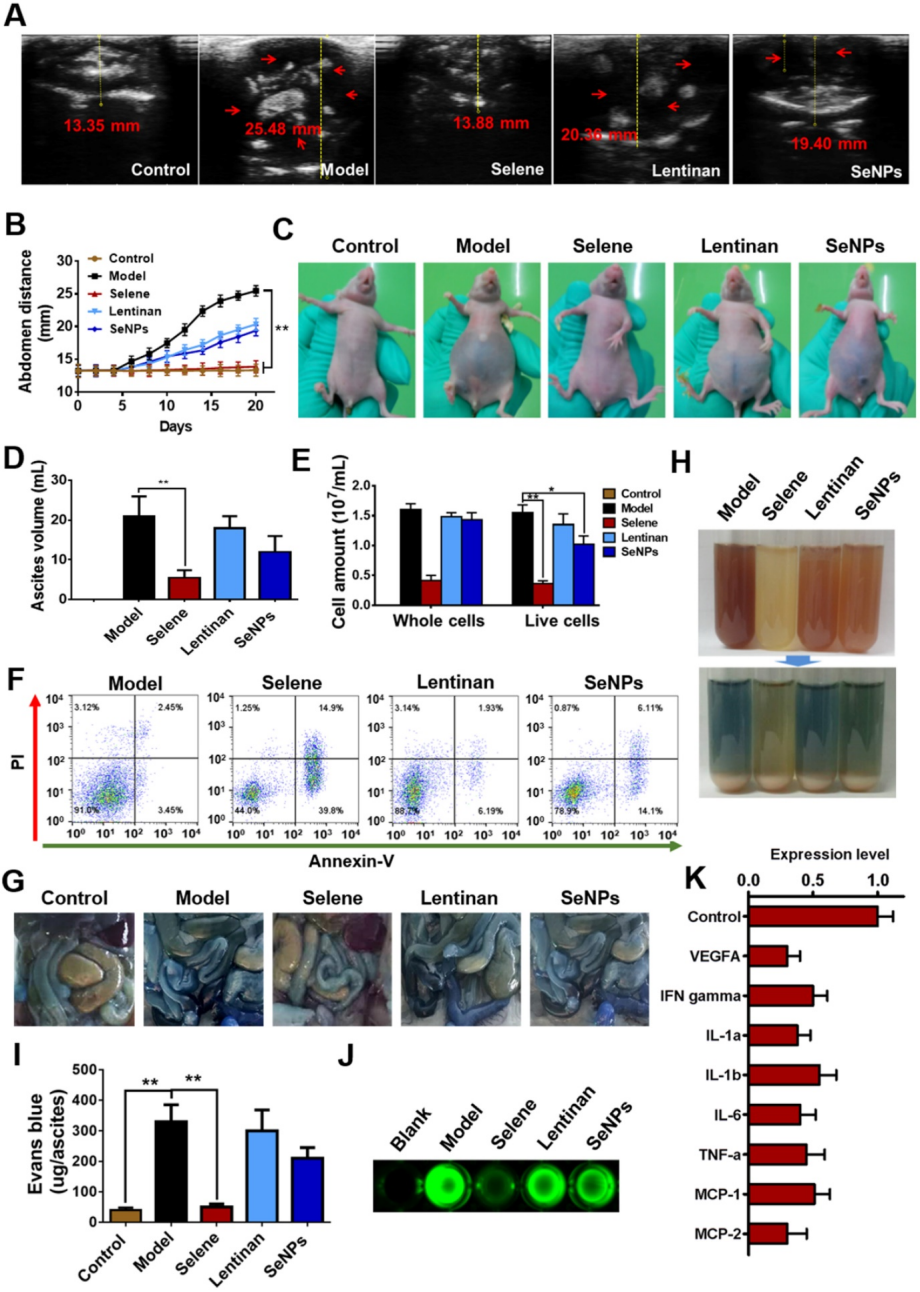
** Selene inhibited ascites induced by ovarian cancer cells *in vivo*. A and B.** Results of B ultrasound and abdominal volume of ascites induced by ovarian cancer cells (OVCAR-3) in different groups. **C.** Representative images of mice in different groups. **D and E.** Volume of ascites and cancer cell numbers in ascites. **F.** Results of cell apoptosis detection of the cancer cells in ascites. **G.** The photographs of Evans blue dye leakage in the intestines and abdominal walls. **H.** The photographs of the aspirated ascites before (upper panel) and after (lower panel) centrifugation. **I.** Comparison of Evans blue amounts in ascites of each group. **J.** The photographs of the FITC-dextran intensity in 20 mL of ascites of each sample. **K.** Analysis results of cytokine antibody chip assay of Selene-treated group compared with the model group. Results are shown as means ± SD (*P < 0.05, **P < 0.01).

An orthotopic OVCAR-3 model was established by implanting cells in athymic nude mice to explore the effect of Selene on malignant ascites. B ultrasound was conducted to measure the volume of ascites in the abdominal cavity (Figures 3A and 3B). The volume of ascites and the number of cancer cells were obviously decreased by Selene compared with those in the other groups (Figures 3C-3E). Moreover, Selene could effectively induce apoptosis of cancer cells in the ascites from the abdominal cavity (Figure 3F). Before the mice were sacrificed, Evans blue and FITC-dextran were intravenously administrated to examine vascular leakage in the peritoneal cavities. Evans blue dye was markedly visible in the intestine and abdominal wall of the mice in model group but barely visible in Selene-treated mice (Figure 3G). The ascites in the model group were highly hemorrhagic with high levels of Evans blue, whereas those in the Selene-treated mice were mildly hemorrhagic with low levels of Evans blue (Figures 3H and 3I). The leakage assay results measured using FITC-dextran was similar to that measured using Evans blue (Figure 3J). These results confirmed that Selene could inhibit the ascites production in OVCAR-3 ascites model. The results of cytokine antibody chip assay showed that Selene decreased the expression of several inflammatory cytokines (Figure 3K), such as IL-1β, IL-6, and TNF-α.

### Selene could influence mitochondrial function and induce cell apoptosis

**Figure 4:**
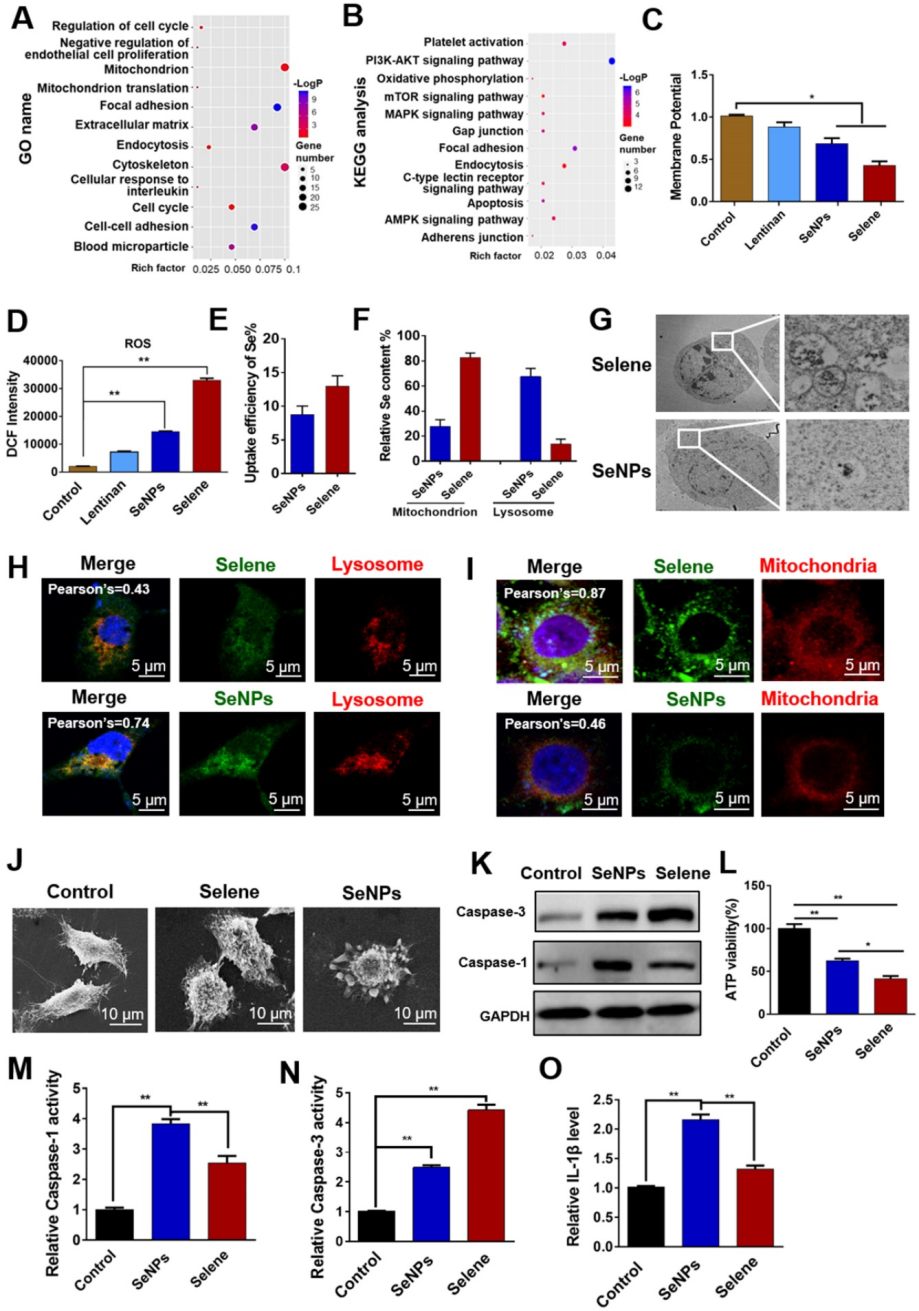
** Selene could influence mitochondrial function and induce apoptosis of OVCAR-3 cells. A and B.** Proteomic analysis results of cells treated with Selene compared with the control group. Differentially expressed proteins were analyzed using GO and KEGG databases to explore the main functions and signaling pathways affected by Selene. **C and D.** Detection results of mitochondrial membrane potential and ROS in Selene treated cells. **E.** Uptake efficiency of Selene and SeNPs in OVCAR-3 cells. **F.** Relative Se content in the mitochondria and lysosomes of OVCAR-3 cells. **G.** Ultrathin sectioning of cells detected by TEM. H. Colocation results of Selene/SeNPs and lysosomes. I. Colocation results of Selene/SeNPs and mitochondria. **J.** SEM images of OVCAR-3 cells. **K.** Expression of caspase 1 and 3 detected by Western blot. **L.** Results of ATP level in cells treated with Selene and SeNPs. **M and N.** Caspase 1 and caspase 3 activities in cells treated with Selene and SeNPs. **O.** The content of IL-1β in each group. Results are shown as means ± SD (*P < 0.05, **P < 0.01).

Proteomic analysis was performed to evaluate the differentially expressed proteins (|logFC| > 1.5) in the Selene-treated groups and study the effect of Selene on the main function and signaling pathway of OVCAR-3 cells. The differential proteins were analyzed using GO and KEGG enrichment analysis. As shown in Figures 4A and 4B, KEGG and GO analysis revealed that the main functions and signaling pathways affected by Selene including oxidative phosphorylation, endocytosis, apoptosis, adhesion, mitochondrion, and mitochondrion translation. The effect of Selene on mitochondrial function was determined on the basis of the above results and the essential role of mitochondria in apoptosis and cell metabolism. The mitochondrial membrane potential decreased after Selene treatment (Figure 4C). Given that mitochondria are the main organelles of ROS production and ROS is closely related to cell apoptosis, the ROS levels were also examined using the DCFH-DA probe in cells treated with Selene, LNT, and SeNPs under the same molar dose. ROS test results showed that Selene could increase the level of ROS (Figure 4D). Furthermore, the uptake efficiency of Selene in cells was analyzed. The Se content in the whole cells, mitochondria, and lysosome was analyzed using ICP-MS. The results showed that the uptake efficiency of Selene was higher than that of SeNPs (Figure 4E). In the Selene-treated cells, Se was enriched in the mitochondria. However, Se was enriched in the lysosome in the SeNPs-treated group (Figure 4F). Ultrathin sectioning and IF (6-coumarin-labeled Selene and SeNPs) results also showed that Selene was mainly enriched in the mitochondria (Figures 4G-4I), whereas SeNPs were mainly enriched in the lysosome. Cell morphological features were characterized using scanning electron microscopy. As shown in Figure 4J, Selene could induce cell shrinkage and apoptotic body formation, which was the specific morphological change in apoptosis. SeNPs caused cell swelling and membrane rupture, which was the specific morphological change in pyroptosis. The caspase-3 (key enzyme in apoptosis) and caspase-1 (key enzyme in pyroptosis) activities were determined to further verify the effect of Selene on cell apoptosis and SeNPs on cell pyroptosis. The results showed that Selene increased the caspase-3 expression (Figure 4K), decreased the ATP levels (Figure 4L), and increased the caspase-3 activity (Figure 4N) in OVCAR-3 cells, and the effect was better than that of SeNPs. However, Selene showed a weaker effect on regulating caspase-1 activity (Figure 4M) and IL-1β content than SeNPs (Figure 4O). These results showed that Selene mainly induced cell apoptosis and SeNPs mainly induced cell pyroptosis.

### Selene targeted mitochondria via caveolae-mediated endocytosis

**Figure 5:**
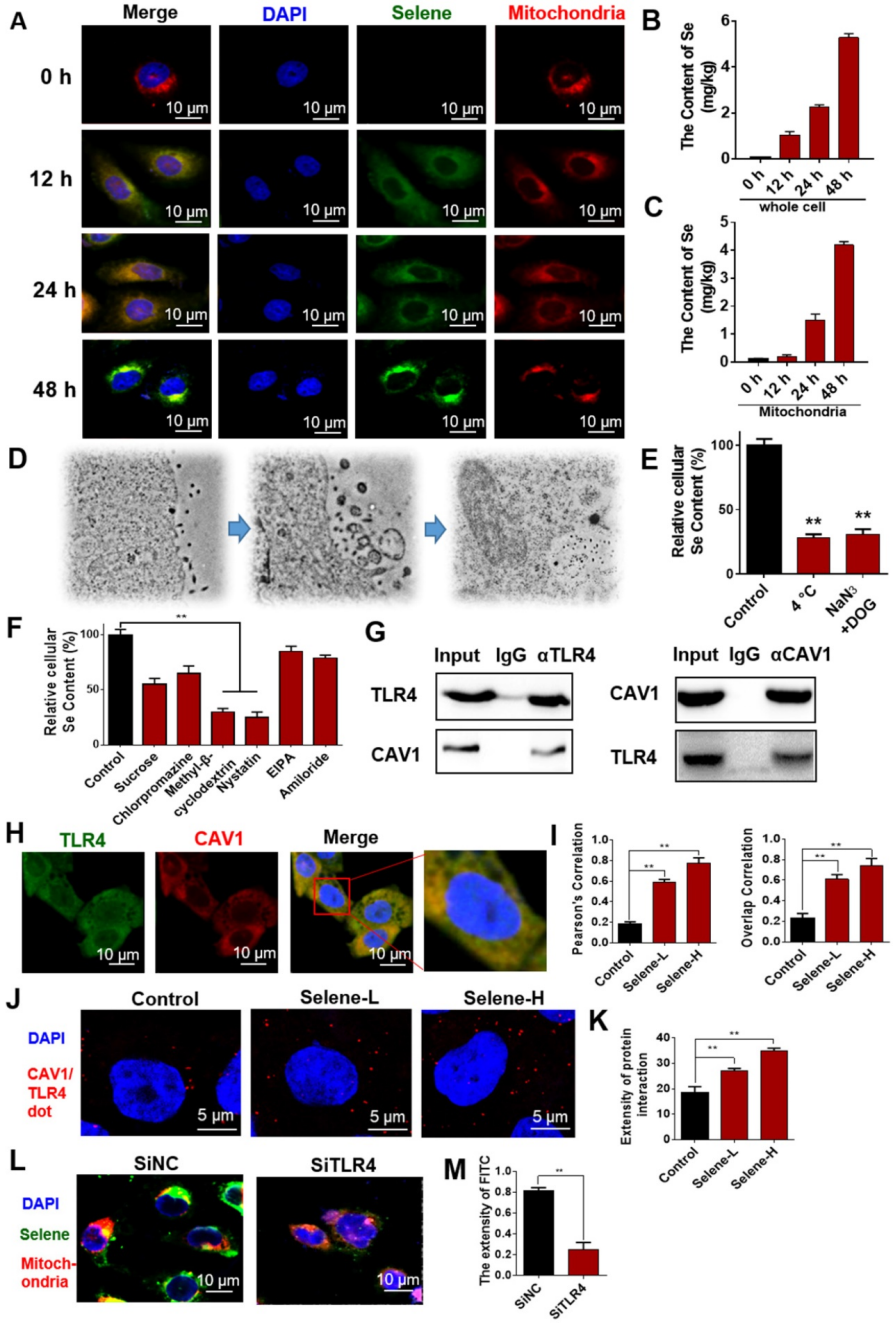
** Selene targeting the mitochondria via the caveolae-mediated endocytosis pathway. A.** Immunofluorescence results of Selene accumulation in cells at different time points. **B and C.** Se content in the whole cell and mitochondria detected using ICP-MS. **D.** Results of ultrathin sectioning of cells treated with Selene detected by the TEM. **E.** Se content in cells after low-temperature (4 °C) and energy-depletion agent (NaN_3_ + deoxyglucose) treatment. **F.** Se content in cells treated with clathrin-mediated endocytic inhibitors (sucrose and chlorpromazine), caveolae-mediated endocytic inhibitors (MβCD and nystatin), and macropinocytosis inhibitor (EIPA and amiloride). **G.** Co-IP results of TLR4 and CAV1 detected by Western blot. **H and I.** Immunofluorescence double-labeling results of TLR4 and CAV1. **J and K.** Duolink PLA results of TLR4 and CAV1 (Selene-L 10 µM, Selene-H 15 µM). **L and M.** Colocation results of Selene and the mitochondria after SiTLR4 treatment, as detected via immunofluorescence. Results are shown as means ± SD (*P < 0.05, **P < 0.01).

To study the dynamic process of Selene entry into the cells, the drug-treated cells were observed at different time points via immunofluorescent microscopy. IF results showed increased accumulation of Selene in the mitochondria over time (Figure 5A). ICP-MS analysis results also showed that the Se content increased in the whole cell and mitochondria (Figures 5B and 5C) over time. Ultrathin sectioning via TEM demonstrated that Selene may be taken up by tumor cells through endocytosis (Figure 5D). An endocytic inhibition experiment was conducted to reveal the mechanism of Selene entry into the cells. As shown in Figure 5E, treatment with NaN_3_ and DOG or at a low temperature of 4 °C instead of 37 °C markedly reduced the cellular uptake of Selene, indicating that Selene entered the cells via energy-dependent endocytosis. Clathrin-mediated endocytic inhibitors (sucrose and chlorpromazine hydrochloride), caveolae-mediated endocytic inhibitors (methyl-β-cyclodextrin and nystatin) and pinocytic inhibitors (EIPA and amiloride) were also used to identify the endocytic mechanism responsible for Selene uptake. As shown in Figure 5F, the specific inhibitors of caveolae-mediated endocytosis could strongly decrease Selene endocytosis, thereby suggesting that the main pathway of Selene entry into cells was caveolae-mediated endocytosis.

### Selene entered cells via TLR4/CAV1 pathway

LNT could inhibit inflammatory cell infiltration by targeting TLR4. CAV1 mainly exists in endocytic vesicles mediated by caveolin. Thus, the LNT in Selene may be involved in caveolae-mediated endocytosis via targeting TLR4. TLR4 may have interaction with CAV1 and mediated the endocytosis of Selene. Co-IP experiments were performed to explore the relationship between TLR4 and CAV1, and the results showed that TLR4 and CAV1 could be co-immunoprecipitated with each other (Figure 5G). IF double-labeling experiments and Duolink PLA also confirmed the co-localization of the two proteins (Figures 5H-K). When TLR4 was knocked down, Selene accumulation in the mitochondria decreased (Figures 5L and 5M). These results indicated that TLR4 participated in the process of Selene entry into the cells and the mitochondria.

### Selene was specifically enriched in the mitochondria through TLR4/TRAF3/MFN1 mediated membrane fusion

**Figure 6:**
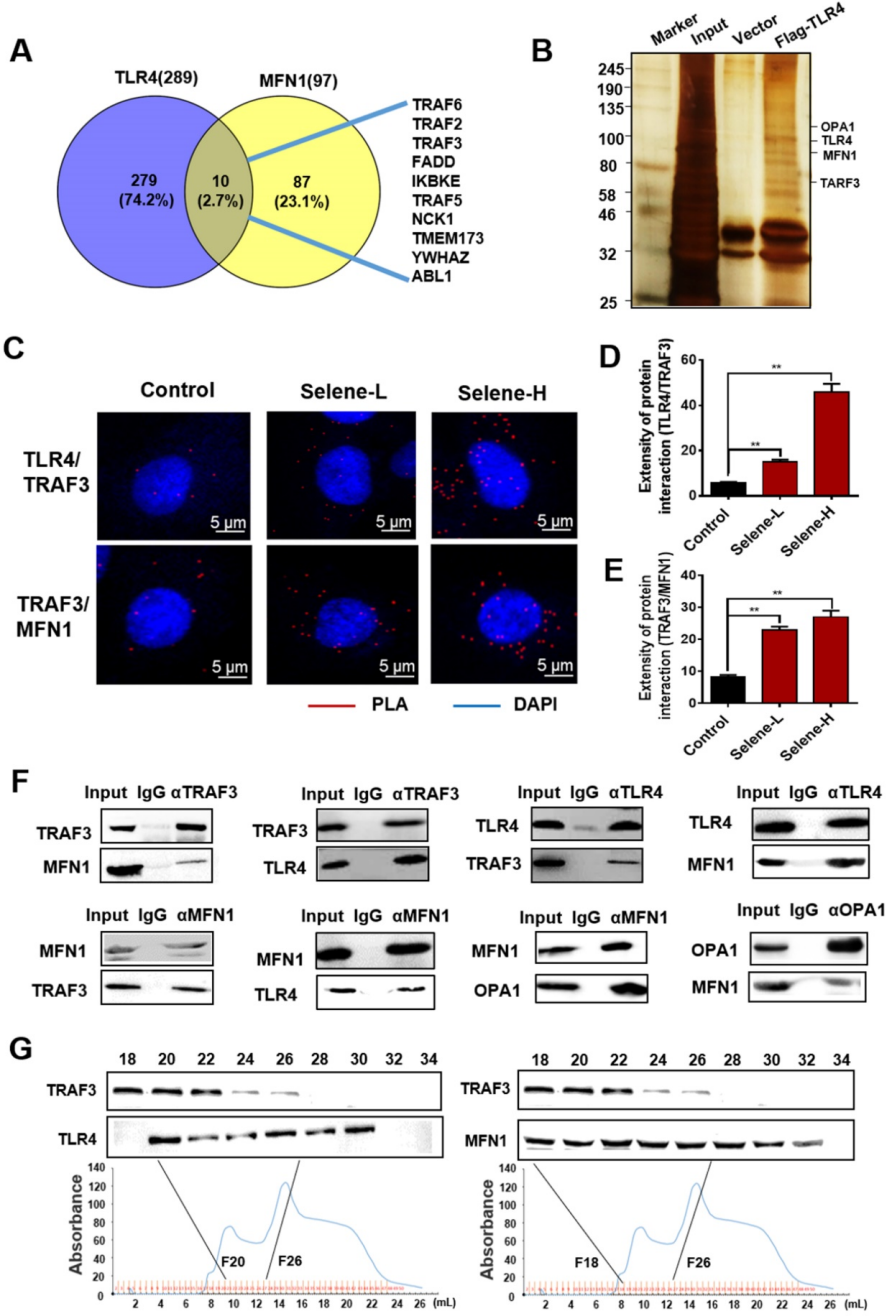
** Selene was specifically enriched in the mitochondria through TLR4/TRAF3/MFN1 protein complex-mediated membrane fusion. A.** Results of potential bridge-linked protein analysis from FpClass database. **B.** Pull-down assay results (IP by the anti-Flag beads) of the input, control vector, and Flag-TLR4 overexpression groups detected via silver staining to identify proteins interacting with TLR4. **C-E.** Duolink PLA results of TLR4/TRAF3 and TRAF3/MFN1 after Selene treatment (Selene-L 10 µM, Selene-H 15 µM). **F.** Whole-cell lysates from OVCAR-3 were immunoprecipitated by anti-TRAF3, anti-TLR4, anti-MFN1, and anti-OPA1 antibodies, followed by Western blot with antibodies against the indicated proteins to verify the interactions of proteins. **G.** Cell lysate was separated by FPLC. Then, TRAF3, TLR4, and MFN1 in the fractions were detected using Western blot. Results are shown as means ± SD (*P < 0.05, **P < 0.01).

The molecular mechanism of Selene entering into the mitochondria was further studied. The above results showed that Selene entered the cells through the caveolae-mediated endocytosis pathway by targeting TLR4. In addition, Selene was mainly enriched in the mitochondria, while SeNPs were mainly enriched in the lysosome, indicating that TLR4 may mediate the process of Selene entering into the mitochondria. The Selene in endocytic vesicles could enter the mitochondria via mitochondrial membrane fusion, as shown in the results of ultrathin sectioning via TEM (Figure 4G). MFN1/OPA1 were the main proteins mediating the mitochondrial membrane fusion 29. The predicted results from FpClass (http://dcv.uhnres.utoronto.ca/FPCLASS/ppis/) showed no direct interaction between TLR4 and MFN1. Thus, a bridge-linked protein between TLR4 and MFN1 must exist. Figure 6A shows the potential bridge-linked protein predicted by FpClass. Flag-TLR4 overexpressed cells were used in the pull-down assay to identify the proteins interacting with TLR4. Silver staining results showed that TRAF3 and mitochondrial membrane fusion proteins MFN1 and OPA1 could co-precipitate with TLR4 (Figure 6B). Duolink PLA experiment confirmed that the interaction between TLR4/TRAF3 and TRAF3/MFN1 could be enhanced by Selene (Figures 6C-6E). Co-IP experiment was performed in OVCAR-3 cells to confirm the interaction. The results showed that TRAF3 could effectively co-immunoprecipitate with TLR4 and MFN1. MFN1 could also effectively co-immunoprecipitate with TRAF3 and TLR4 (Figure 6F). Fast protein liquid chromatography (FPLC) was used to separate the cell extract, and the results showed that TRAF3 may have an interaction with TLR4 and MFN1 (Figure 6G). The molecular docking simulation and scoring were also carried out (https://cluspro.org/) to verify the interaction of TLR4/TRAF3 and TRAF3/MFN1. And the amino acids involved in the interaction interface were analyzed, which show that there are hydrogen bond interaction and van der Waals force between proteins (Figure S2 in supplementary materials). The results showed that the weighted score of TLR4/TRAF3 was -969.9 and the lowest energy was -992.1. The weighted score of TRAF3/MFN1 was -975.8 and the lowest energy was -975.8. These results further verified the interaction of TLR4/TRAF3 and TRAF3/MFN1.

**Figure 7:**
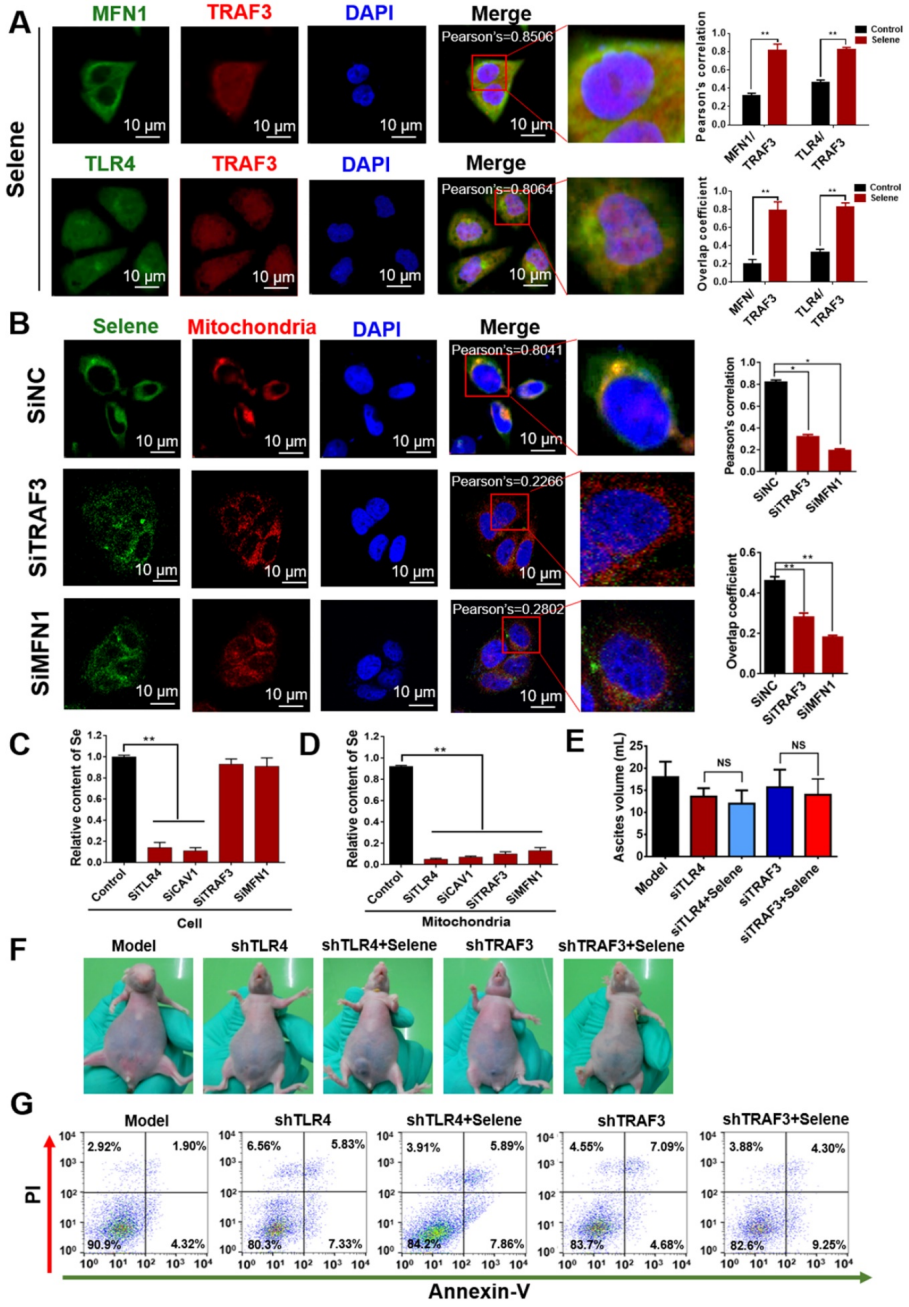
** Selene was specifically enriched in the mitochondria via TLR4/TRAF3/MFN1 pathway. A.** Colocalization of MFN1/TRAF3 and TLR4/TRAF3 was analyzed by immunostaining of OVCAR-3 cells and detected using confocal microscopy. **B.** Colocation of Selene and mitochondria in OVCAR-3 cells after TRAF3 and MFN1 knockdown. **C and D.** Se content in the whole cells and mitochondria as detected using ICP-MS in TRAF3 and MFN1 knocked down OVCAR-3 cells. **E and F.** The effect of Selene on OVCAR-3 ascites model when TLR4 and TRAF3 in OVCAR-3 cells were knocked down. **G.** Results of cell apoptosis detection in the OVCAR-3 ascites model when TLR4 and TRAF3 in OVCAR-3 cells were knocked down. Results are shown as means ± SD (*P < 0.05, **P < 0.01).

**Figure 8:**
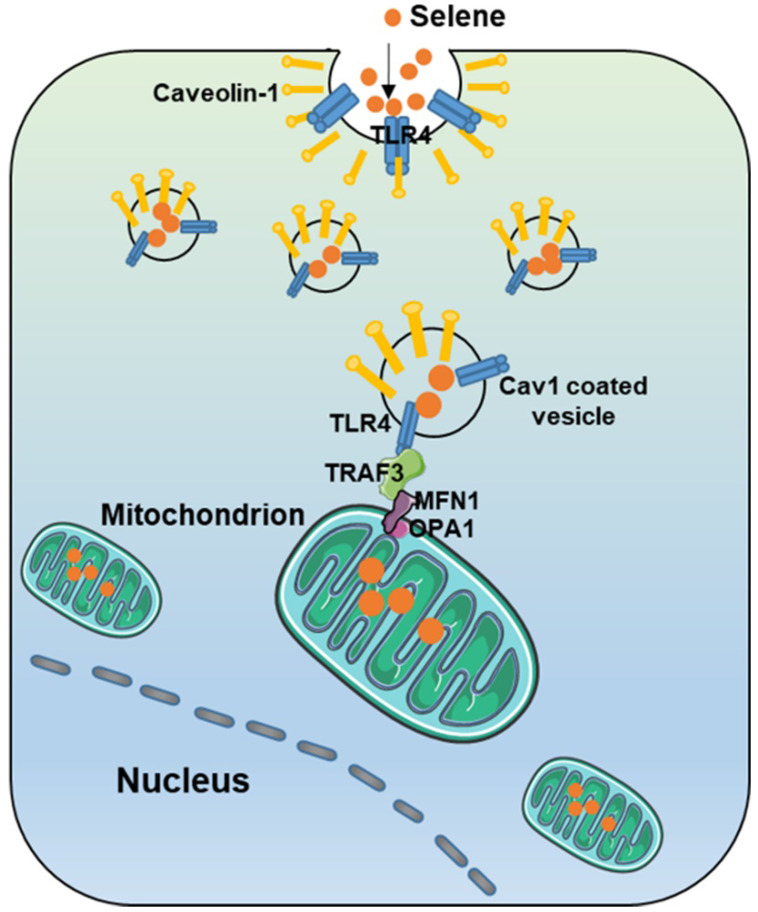
Model of Selene targeting the mitochondria via TLR4/TRAF3/MFN1 pathway.

As shown in Figure 7A, Selene promoted the colocalization of MFN1 and TLR4 with TRAF3. Knocking down the expression levels of TRAF3 and MFN1 could decrease Selene accumulation in cells and colocalization with the mitochondria (Figure 7B). The Se content was tested using ICP-MS, and the results showed that it decreased in the whole cell and the mitochondria under TLR4/CAV1 siRNA treatment. The Se content in the mitochondria was decreased in the TRAF3/MFN1 siRNA-treated cells. However, no obvious changes of the Se content in the whole cell was observed under TRAF3/MFN1 siRNA treatment, indicating that TRAF3/MFN1 mainly affected the process of Selene entering into the mitochondria (Figures 7C and 7D). The* in vivo* experiment showed that Selene exhibited minimal effects on suppressing ascites and inducing tumor cell apoptosis when TLR4 and TRAF3 were knocked down (Figures 7E-7G). In conclusion, the TLR4/TRAF3/MFN1 pathway allowed Selene to target the mitochondria (Figure 8).

## Discussion

Malignant ascites is a sign of peritoneal carcinomatosis, which indicates the presence of malignant cells in the peritoneal cavity. The survival of patients with malignant ascites is poor 30, 31. In addition, malignant ascites has a significant effect on the quality of life. Current treatment cannot effectively improve the overall survival but may improve the life quality of patients. Paracentesis is effective in providing temporary symptom relief but requires frequent repeated procedures. Diuretics typically do not work well for malignant ascites. The options for durable symptom management include i.p. catumaxomab and hyperthermic i.p. chemotherapy. The current chemotherapy cannot suppress inflammatory cytokines effectively and may lead to recurrence of ascites. Se is an essential and unique trace element with multiple biological functions. As therapeutic agents for cancer and other diseases, different forms of Se exist, including inorganic and organic Se compounds and SeNPs, which possess excellent biological activities and low toxicity, especially the functionalized SeNPs 20. In the present study, Selene was prepared using Na_2_SeO_3_ and LNT under reduction conditions. Selene differed from elemental SeNPs in terms of chemical composition and bio-efficacy. Selene exhibited relatively better therapeutic effects for malignant ascites induced by ovarian cancer and EAC. Selene could decrease the volume of ascites and the number of cancer cells. Selene could also effectively induce apoptosis of cancer cells in ascites and inhibit the inflammatory cytokines content in the ascites. Compared with Selene, SeNPs showed no obvious effect on malignant ascites. Selene could inhibit vascular leakage in the peritoneal cavities. Pharmacological research showed that Selene specifically targets the mitochondria, thereby inducing tumor cell apoptosis.

Nanomaterials are usually ingested by cells through endocytosis 32. Different types of endocytosis, including phagocytosis, macropinocytosis, and clathrin/caveolae mediated endocytosis 33, are involved in the uptake of nanomaterials. Receptor mediated endocytosis is the most effective method for nanodrug intake 34. Ligands on the nanoparticles surface bind with cell-surface receptors, thereby inducing cell membrane invagination to encapsulate the nanoparticles 35. The field of surface-decorated SeNPs has gained more attention than that of non-functionalized elemental SeNPs. The surfaces of SeNPs decorated with *Polyporus rhinocerus* polysaccharides, folate, sialic acid, and chitosan have been developed 36-39. The rationale behind this conjugation is the ability of the decorated ligand to target membrane receptors/transporters expressed in cancer cells. In the present study, the inhibitors of macropinocytosis and clathrin/caveolae-mediated endocytosis were used to identify the endocytic mechanism responsible for Selene uptake. The results confirmed that Selene was consumed by tumor cells through caveolae-mediated endocytosis. In Selene, the -OH groups of LNT interacted with SeNPs. LNT could disperse SeNPs and enhance the targeting of SeNPs to tumor cells. LNT could also exert its efficacy by targeting TLR4 14, 22. The results of the present study revealed the interaction between TLR4 and CAV1. Selene could target TLR4, which could then induce cell membrane invagination. These findings indicated that TLR4 could act as a specific cellular receptor mediating caveolae-mediated endocytosis to ingest Selene.

Drugs delivery to the mitochondria is a useful strategy in drug research 40. The method widely used to deliver drugs targeting the mitochondria is covalent linking of a triphenylphosphonium (TPP) moiety to a pharmacophore of interest 41. The advantages of TPP+-based mitochondrial targeting method include stability in biological systems, a combination of hydrophilic and lipophilic properties, and relatively simple purification. However, TPP+-based mitochondrial targeting has limited application because of poor biocompatibility, solubility, and clinical application of therapeutic agents. Therefore, the use of nanocarriers may enhance the potential of therapeutic agents 42. In the present study, compared with elemental SeNPs, Selene was mainly located in the mitochondria, indicating that LNT may mediate the targeted distribution of Selene in cells. The results of ultrathin sectioning via TEM showed that Selene was taken up by the mitochondria instead of lysosomes through membrane fusion. Thus, the endocytic vesicles formed by Selene and TLR4/CAV1 could enter the mitochondria through membrane fusion. MFN1 is a key protein during mitochondrial membrane fusion 29. Given that TLR4 is not usually located in the mitochondrial membrane, a bridge protein between TLR4 and MFN1 possibly exist to mediate the membrane fusion process of endocytic vesicle to the mitochondria. The PPI prediction from FpClass database and the results of pull-down, Co-IP, and Duolink assays showed that TRAF3 mediated the interaction between TLR4 and MFN1. These results indicated that TLR4/TRAF3/MFN1 was a specific nano-drug delivery pathway targeting the mitochondria.

Tumor cells can excrete many types of cytokines, such as VEGFA and TNF-α, thereby altering the permeability of microvascular networks and causing transudative and exudative ascites 43, 44. Macrophage and inflammatory cells could induce pyroptosis or necroptosis, release several necrotic materials and inflammatory mediators, and increase the volume of ascites 8, 45. Pyroptosis is a form of necrotic and inflammatory programmed-cell death with a strong inflammatory reaction. The present study showed that elemental SeNPs were mainly ingested by the lysosome and caused lysosome breakdown. Lysosome contents, particularly cathepsin B, could mediate pro-inflammatory response 46. The present study also revealed that elemental SeNPs induced pyroptosis and the release of inflammatory factor. Instead of the lysosome, Selene was mainly taken up by the mitochondria. Selene could interfere with mitochondrial function and decrease the mitochondrial membrane potential. Moreover, Selene induced the caspase9/caspase3 apoptotic pathway and decreased the inflammatory factor in ascites.

In conclusion, Selene could target the mitochondria of tumor cells via the TLR4/TRAF3/MFN1 pathway, induce tumor cell apoptosis, and inhibit inflammatory cytokine secretion in ascites. Selene is effective for treating malignant ascites and may be developed as a drug for malignant ascites treatment in clinics. This work showed that TLR4/TRAF3/MFN1 is a mitochondrial targeting molecular pathway for nanoparticles, which provides a theoretical basis for the development of mitochondrion-targeted anticancer nanodrugs.

## Supplementary Material

Supplementary figures.
