# Lentinan-functionalized Selenium Nanoparticles target Tumor Cell Mitochondria via TLR4/TRAF3/MFN1 pathway: Erratum

**DOI:** 10.7150/thno.78949

**Published:** 2022-11-02

**Authors:** Hui-Juan Liu, Yuan Qin, Zi-Han Zhao, Yang Zhang, Jia-Huan Yang, Deng-Hui Zhai, Fang Cui, Ce Luo, Man-Xi Lu, Piao-Piao Liu, Heng-Wei Xu, Kun Li, Bo Sun, Shuang Chen, Hong-Gang Zhou, Cheng Yang, Tao Sun

**Affiliations:** 1 State Key Laboratory of Medicinal Chemical Biology and College of Pharmacy, Nankai University, Tianjin, China.; 2 Tianjin Key Laboratory of Early Druggability Evaluation of Innovative Drugs and Tianjin Key Laboratory of Molecular Drug Research, Tianjin International Joint Academy of Biomedicine, Tianjin, China.; 3 Department of Gastroenterology and Hepatology, General Hospital, Tianjin Medical University, Tianjin Institute of Digestive Disease, Tianjin, China.; 4 Department of Anesthesiology, Tianjin Fourth Central Hospital, Tianjin, China.

The authors apologize that the original version of the above article contains errors that need to be corrected. Incorrect images for Figure 3G were used during figure assembly. The correction does not affect the conclusions of the above paper. The authors sincerely apologize for any inconvenience these errors may have caused. The corrected Figure 3G appears below.

## Figures and Tables

**Figure 3G:**
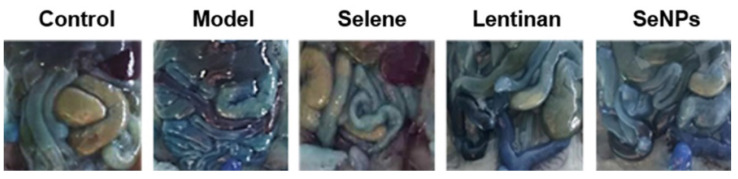
Corrected figure.

