# Supplementary material for: Lentinan-functionalized Selenium Nanoparticles target Tumor Cell Mitochondria via TLR4/TRAF3/MFN1 pathway
Source: Theranostics. 2020 Jul 11;10(20):9083–99. doi: 10.7150/thno.46467 (PMC7415812; doi:10.7150/thno.46467)

Supplementary Materials for

Lentinan-functionalized selenium nanoparticles target tumor cell mitochondria via TLR4/TRAF3/MFN1 pathway

Hui-Juan Liu[#,1,2]*, Yuan Qin[1,2]*, Zi-Han Zhao[1,2]*, Yang Zhang[4]*, Jia-Huan Yang[1,2], Deng-Hui Zhai[1,2], Fang Cui[1,2], Ce Luo[1], Man-Xi Lu[1], Piao-Piao Liu[1], Heng-Wei Xu[1,2], Kun Li[1,2], Bo Sun[2], Shuang Chen[2], Hong-Gang Zhou[#1], Cheng Yang[#,1,2], Tao Sun[#,1,2,3]

[1]State Key Laboratory of Medicinal Chemical Biology and College of Pharmacy, Nankai University, Tianjin, China

[2]Tianjin Key Laboratory of Early Druggability Evaluation of Innovative Drugs and Tianjin Key Laboratory of Molecular Drug Research, Tianjin International Joint Academy of Biomedicine, Tianjin, China

[3]Department of Gastroenterology and Hepatology, General Hospital, Tianjin Medical University, Tianjin Institute of Digestive Disease, Tianjin, China.

[4]Department of Anesthesiology, Tianjin Fourth Central Hospital, Tianjin, China

*These authors have contributed equally to this work

[#]Correspondence to: Tao Sun, email: tao.sun@nankai.edu.cn

Cheng Yang, email: cheng.yang@nankai.edu.cn

Hong-gang Zhou, email: honggang.zhou@nankai.edu.cn

` Hui-juan Liu, email: liuhuijuanxyz@163.com

This PDF file includes

Figs. S1-S3

Figure S1

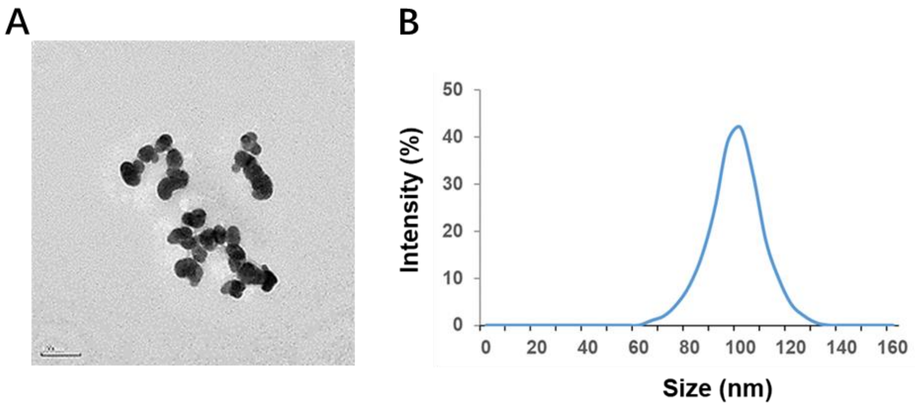

Figure S1. The characterization of SeNPs. (A) The TEM picture of SeNPs; (B) Particle size distribution of SeNPs.

Figure S2

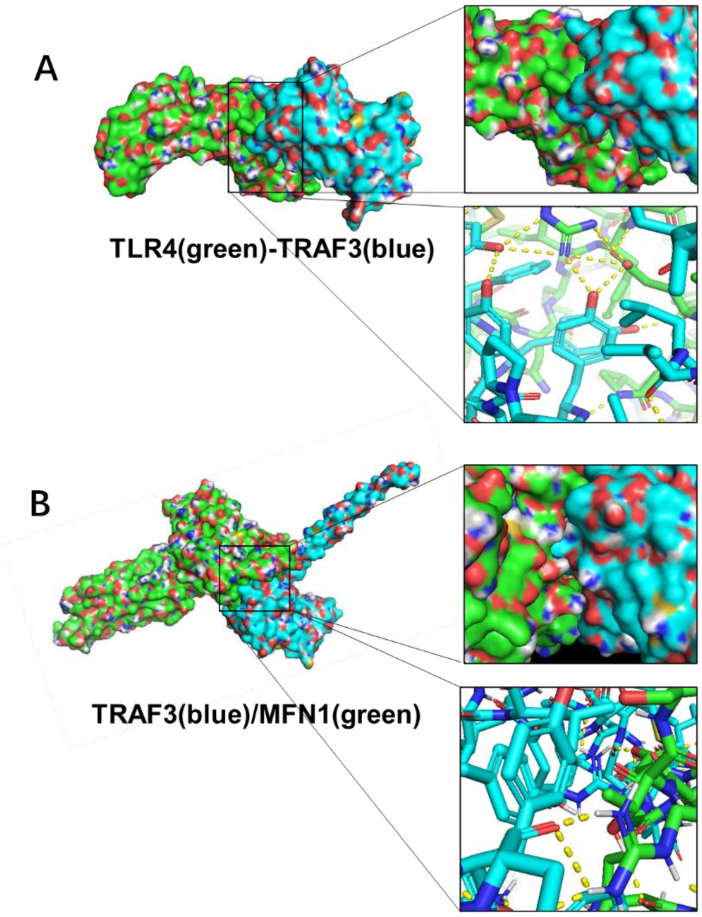

Figure S2. The molecular docking results of TLR4/TRAF3 (A) and TLR4/TRAF3 (B) (https://cluspro.org/).

Figure S3. The uncropped whole membrane western blot for Figure 4J, Figure 5G, Figure 6F, and Figure 6G.

Figure. 4J

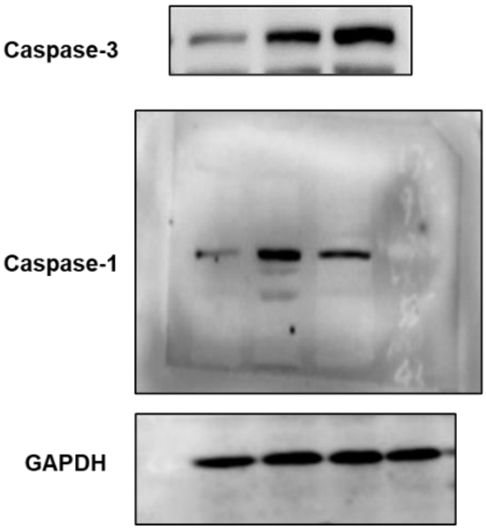

Figure. 5G

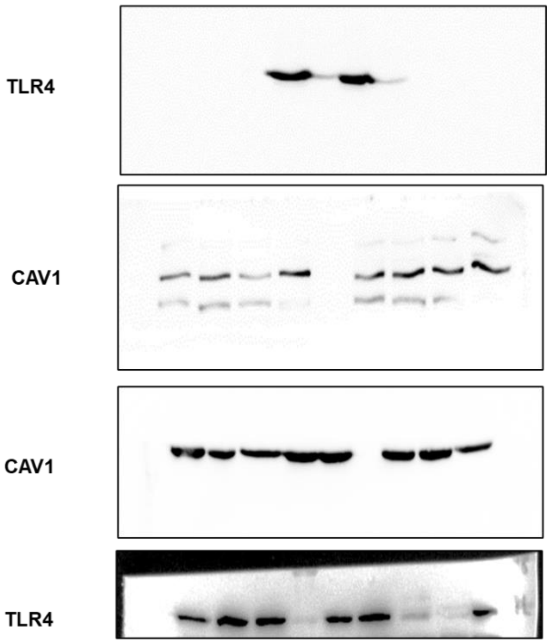

Figure. 6F

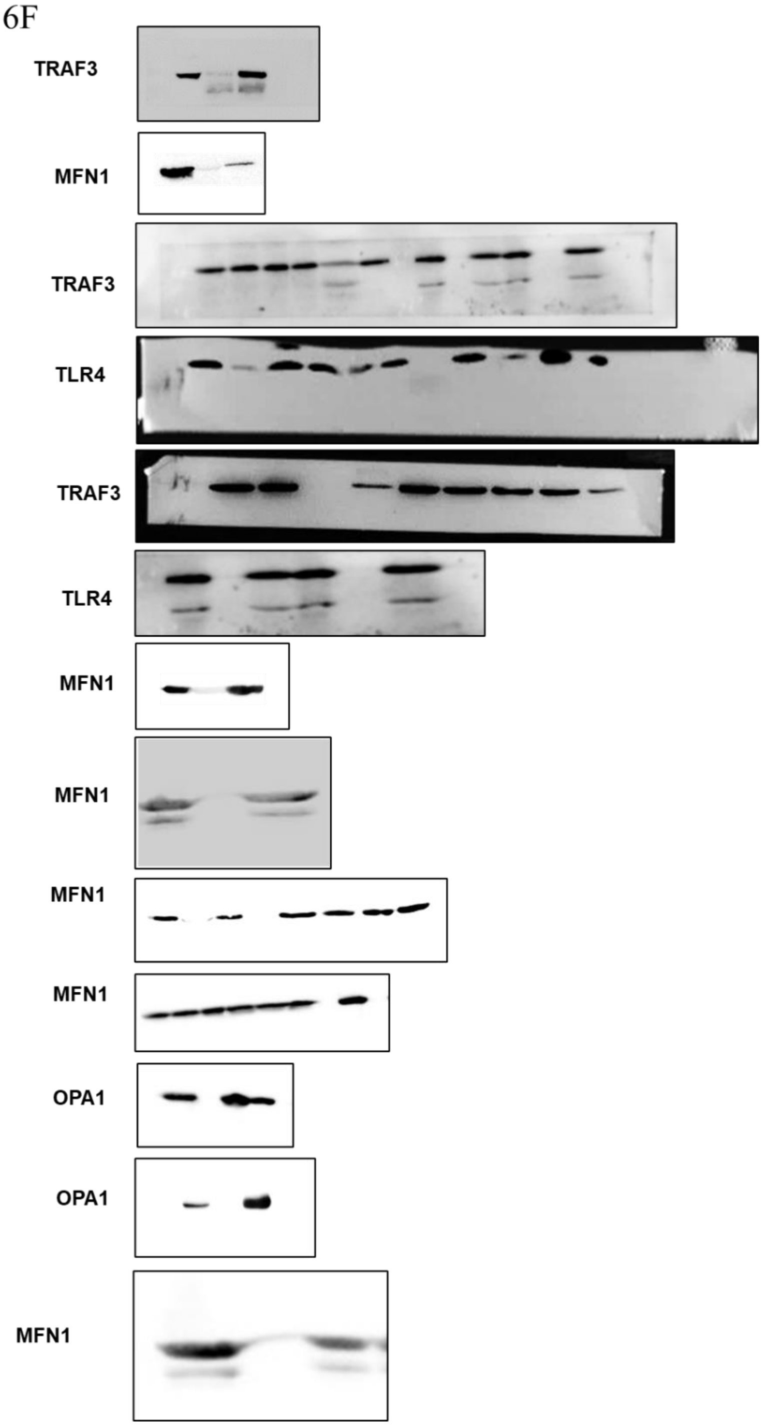

TRAF3
MFN1
TRAF3
TLR4
TRAF3
TLR4
MFN1
MFN1
MFN1
MFN1
OPA1
OPA1
MFN1

Figure. 6G

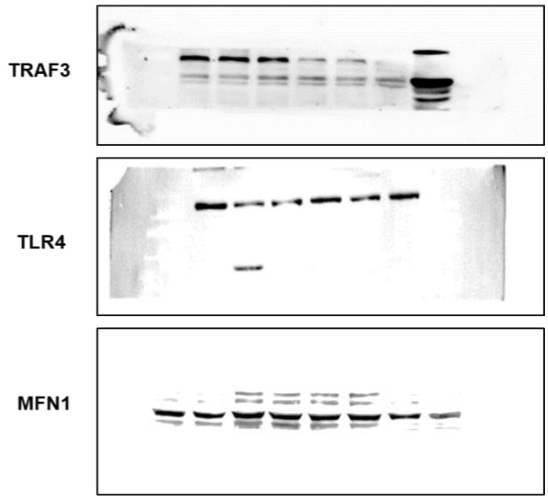

Supplement: Supplementary file 1 — Supplementary figures. [file thnov10p9083s1.pdf]
